# Analytical Solutions of a Modified Predator-Prey Model through a New Ecological Interaction

**DOI:** 10.1155/2019/4849393

**Published:** 2019-10-16

**Authors:** Noufe H. Aljahdaly, Manar A. Alqudah

**Affiliations:** ^1^Department of Mathematics, Faculty of Sciences and Arts-Rabigh Campus, King Abdulaziz University, Jeddah, Saudi Arabia; ^2^Mathematical Science Department, Princess Nourah Bint Abdulrahman University, P.O. Box 84428, Riyadh 11671, Saudi Arabia

## Abstract

The predator-prey model is a common tool that researchers develop continuously to predict the dynamics of the animal population within a certain phenomenon. Due to the sexual interaction of the predator in the mating period, the males and females feed together on one or more preys. This scenario describes the ecological interaction between two predators and one prey. In this study, the nonlinear diffusive predator-prey model is presented where this type of interaction is accounted for. The influence of this interaction on the population of predators and preys is predicted through analytical solutions of the dynamical system. The solutions are obtained by using two reliable and simple methods and are presented in terms of hyperbolic functions. In addition, the biological relevance of the solutions is discussed.

## 1. Introduction

Many phenomena in natural science, biology, physics, or engineering are studied by developing a mathematical model that consists of partial differential equations. A prey-predator system is a well-known mathematical model for studying the species' population and density [1, 2]. Many different interactions in this model are very significant phenomena in nature's population [3, 4]. Alqudah [5] modified the diffusive predator-prey model in which two predators interact with one or more preys in the mating period as follows:

(1) $$\begin{array}{c} u_{t}-D_{1}\left(\Delta u\right)=a_{1}u-a_{2}u^{2}-a_{3}uv-a_{4}uv^{2},\\ v_{t}-D_{2}\left(\Delta v\right)=a_{3}uv-a_{5}v-a_{6}v^{2}+a_{4}uv^{2}. \end{array}$$

The biological meaning of each term is presented in Table 1. We assume that *a*_*i*_ for all *i*=1,2,…, 6 are positive constants and *D*=*D*_1_/*D*_2_, *D*_1_=0,  *D*_2_ ≠ 0 which means that the predator is moving toward the prey.

This model focuses on the term *uv* ^2^ which refers to a positive interaction with respect to the predator species, like sexual interaction in the mating period, to proliferate the predators. Thus, the males and females are together and feed on the same prey or more, but this term is negative with respect to the prey. In [5], the existence and the uniqueness of the solution of model (1) are studied, and the scaling is introduced to get the dimensionless parameters. Thus, model (1) can be expressed as

(2) $$\begin{array}{c} U_{t}=U-U^{2}-UV-UV^{2},\\ V_{t}=V_{XX}-k_{2}k_{1}V-k_{1}V^{2}+k_{1}VU+k_{1}UV^{2}, \end{array}$$

where $k_{1}=\sqrt{a_{4}a_{1}}/a_{2}$ and *k*_2_=*a*_5_/(*a*_1_ *k*_1_).

One way to understand the applications and dynamic system is finding the analytical or approximate solutions. In the field of mathematical computation, several methods have been developed to find the analytical solutions such as the tanh method [6], the Riccati equation expansion method [7], the (*G*′/*G* ^2^)-expansion method [8, 9], the algebraic method [10], and so on. In this paper, the exact and stable solutions of the system are obtained using two sufficient methods: (i) the (*G*′/*G*)-expansion method [11] and (ii) the generalized auxiliary equation method [12].

This paper is organized as follows.  Section 2 is the description of two employed methods.  Section 3 presents the analytical solutions of the considered dynamical system.  Section 4 is the discussion of the results, including the biological interpretation. The last section is the conclusion of the work and the results.

## 2. Description of Algorithms

This section shows briefly the steps of applying the function-expansion method for finding analytical solutions. We chose two methods that are simple and reliable using the asymptotic software (MATHEMATICA), which are the (*G*′/*G*)-expansion method and the generalized auxiliary equation method. Let us consider the nonlinear evaluation equation of the form

(3) $$\begin{array}{c} P_{1}\left(U,V,U_{t},V_{t},U_{x},V_{x},U_{xx},V_{xx},\ldots \right)=0,\\ P_{2}\left(U,V,U_{t},V_{t},U_{x},V_{x},U_{xx},V_{xx},\ldots \right)=0, \end{array}$$

where *𝒫*_1_ and *𝒫*_2_ are polynomials in *U*, *V* and their derivatives and *U*(*x*, *t*) and *V*(*x*, *t*) are two unknown functions. The methods that are considered in this article have the common following steps:

- Transforming the function *U*(*x*, *t*) and *V*(*x*, *t*) to *u*_1_(*ξ*) and *v*_1_(*ξ*), respectively, by applying the transformation *ξ*=*x* − *ct*, where *c* is the wave speed. Thus, the system of equations (3) is reduced to the ordinary differential equation (ODE)

(4) $$\begin{array}{c} P_{1}\left(u_{1},v_{1},-cu_{1_{\xi }},-cv_{1_{\xi }},u_{1_{\xi }},v_{1_{\xi }},u_{1_{\xi \xi }},v_{1_{\xi \xi }},\ldots \right)=0,\\ P_{2}\left(u_{1},v_{1},-cu_{1_{\xi }},-cv_{1_{\xi }},u_{1_{\xi }},v_{1_{\xi }},u_{1_{\xi \xi }},v_{1_{\xi \xi }},\ldots \right)=0. \end{array}$$

(ii) Assuming the function *u*_1_(*ξ*) and *v*_1_(*ξ*) are expressed by the following polynomials:

(5) $$\begin{array}{c} u_{1}\left(\xi \right)=\overset{n}{\underset{i=0}{\sum }}\alpha_{i}{\left(F\left(\xi \right)\right)}^{i},\\ v_{1}\left(\xi \right)=\overset{m}{\underset{i=0}{\sum }}\beta_{i}{\left(F\left(\xi \right)\right)}^{i}, \end{array}$$

  where *n* > 0 and *m* > 0 are the degrees of the polynomial of *u*_1_ and *v*_1_, respectively. We obtain the value of *n* and *m* by the homogeneous balance theory [13–15].
(iii) Substituting the polynomials (5) into equation (4) to obtain polynomials of the function *F*(*ξ*).
(iv) Equating the confections of (*F*(*ξ*))^*i*^, *i*=1,2,…, *n* (or *m*) to zero to obtain a system in terms of variables *α*_*i*_, *β*_*i*_ and *c*.
(v) Solving the system to find the value of *α*_*i*_, *β*_*i*_ and *c*.
(vi) Substituting the obtained variables *α*_*i*_, *β*_*i*_ and the value of the function *F*(*ξ*) into equation (5) to construct the solutions of *U* and *V*.

The main difference between the methods utilized in this paper is the value of the function *F*(*ξ*).

1. The (*G*′/*G*)-expansion method where *F*=*G*′/*G* satisfies

(6) $$\begin{array}{c} F^{\prime }=-F^{2}-\lambda F-\mu , \end{array}$$

  and *G* satisfies the second order ODE:

(7) $$\begin{array}{c} G^{\prime \prime }+\lambda G^{\prime }+\mu G=0. \end{array}$$

  The function *F*(*ξ*) is the general solution of the equation (6) as follows:

(8) $$\begin{array}{c} F\left(\xi \right)=\left\{\begin{array}{cc} \frac{\sqrt{\lambda^{2}-4\mu }}{2}\frac{\left(A_{1}\cos\;h\left(\left(1/2\right)\sqrt{\lambda^{2}-4\mu }\xi \right)+A_{2}\sin\;h\left(\left(1/2\right)\sqrt{\lambda^{2}-4\mu }\xi \right)\right)}{\left(A_{1}\sin\;h\left(\left(1/2\right)\sqrt{\lambda^{2}-4\mu }\xi \right)+A_{2}\cos\;h\left(\left(1/2\right)\sqrt{\lambda^{2}-4\mu }\xi \right)\right)}-\frac{\lambda }{2}, & \text{for }\lambda^{2}-4\mu >0,\\ \frac{\sqrt{-\lambda^{2}+4\mu }}{2}\frac{\left(A_{1}\cos\left(\left(1/2\right)\sqrt{-\lambda^{2}+4\mu }\xi \right)-A_{2}\sin\left(\left(1/2\right)\sqrt{-\lambda^{2}+4\mu }\xi \right)\right)}{\left(A_{1}\sin\left(\left(1/2\right)\sqrt{-\lambda^{2}+4\mu }\xi \right)+A_{2}\cos\left(\left(1/2\right)\sqrt{-\lambda^{2}+4\mu }\xi \right)\right)}-\frac{\lambda }{2}, & \text{for }\lambda^{2}-4\mu <0,\\ \frac{A_{2}}{\left(A_{1}+A_{2}\xi \right)}-\frac{\lambda }{2}, & \text{for }\lambda^{2}-4\mu =0. \end{array}\right. \end{array}$$

(2) The generalized auxiliary equation method where *F* satisfies the auxiliary equation

(9) $$\begin{array}{c} F^{\prime }=\sqrt{h_{0}+h_{1}F+h_{2}F^{2}+h_{3}F^{3}+h_{4}F^{4}}. \end{array}$$

 The general solutions of the auxiliary equation (9) have several types of solutions depending on the value of $h_{i}$, i=1,2,…,4. The following are some types of the solution and the reader is referred to [10, 12] to find more cases.
  (I) If $h_{0}=h_{1}=0$, the solution of equation (9) is expressed as follows:

(10) $$\begin{array}{c} F\left(\xi \right)=\left\{\begin{array}{cc} \frac{2h_{2}\sec\;h\left(\sqrt{h_{2}}\xi \right)}{\sqrt{\delta_{1}}-h_{4}\sec\;h\left(\sqrt{h_{2}}\xi \right)}, & h_{2}>0,\;\delta_{1}>0,\;h_{3}=h_{4},\\ \frac{2h_{2}\csc\;h\left(\sqrt{h_{2}}\xi \right)}{\sqrt{-\delta_{1}}-h_{4}\csc\;h\left(\sqrt{h_{2}}\xi \right)}, & h_{2}>0,\;\delta_{1}<0,\;h_{3}=h_{4},\\ -\frac{h_{2}}{h_{4}}\left(1\pm \;\tan\left(\left(\sqrt{h_{2}}/2\right)\xi \right)\right), & h_{2}>0,\;\delta_{1}=0,\\ \frac{h_{2}\sec\;h^{2}\left(\left(1/2\right)\sqrt{h_{2}}x\right)}{2\sqrt{h_{2}h_{4}}\tan\;h\left(\left(1/2\right)\sqrt{h_{2}}x\right)-h_{3}}, & h_{2}>0,\\ \frac{h_{2}\sec^{2}\left(\left(1/2\right)\sqrt{-h_{2}}x\right)}{2\sqrt{-h_{2}h_{4}}\tan\left(\left(1/2\right)\sqrt{-h_{2}}x\right)-h_{3}}, & h_{2}<0. \end{array}\right. \end{array}$$

  where *δ*_1_=*h*_3_^2^ − 4*h*_2_ *h*_4_.
(II) If *h*_0_=*h*_1_=*h*_3_=0,

(11) $$\begin{array}{c} F\left(\xi \right)=\left\{\begin{array}{cc} \sqrt{\left(-h_{2}/h_{4}\right)}\text{sec }h\left(\sqrt{h_{2}}x\right), & h_{2}>0,\;h_{4}<0,\\ \sqrt{\left(-h_{2}/h_{4}\right)}\text{sec}\left(\sqrt{-h_{2}}x\right), & h_{2}<0,\;h_{4}>0,\\ \frac{1}{\sqrt{h_{4}}x}, & h_{2}=0,\;h_{4}>0. \end{array}\right. \end{array}$$

(III) If *h*_0_=*h*_1_=*h*_4_=0,

(12) $$\begin{array}{c} F\left(\xi \right)=\left\{\begin{array}{cc} \left(-h_{2}/h_{3}\right)\sec\;h^{2}\left(\frac{\sqrt{h_{2}}\xi }{2}\right), & h_{2}>0,\\ \left(-h_{2}/h_{3}\right)\sec\;\left(\frac{\sqrt{-h_{2}}\xi }{2}\right), & h_{2}<0,\\ \frac{1}{h_{3}\xi^{2}}, & h_{2}=0. \end{array}\right. \end{array}$$

## 3. Analytical Solutions of the Diffusive Predator-Prey System

In this section, the analytical solutions of model (1) will be found by the methods that are described in the previous section. First, introducing the traveling wave solution *ξ*=*X* − *cT* reduces model (1) as

(13a) $$\begin{array}{c} -cu_{1}^{\prime }=u_{1}-u_{1}^{2}-u_{1}v_{1}-u_{1}v_{1}^{2}, \end{array}$$

(13b) $$\begin{array}{c} -cv_{1}^{\prime }=v_{1}^{\prime \prime }-k_{1}k_{2}v_{1}-k_{1}v_{1}^{2}+k_{1}u_{1}v_{1}+k_{1}u_{1}v_{1}^{2}, \end{array}$$

where *U*(*X*, *T*)=*u*_1_(*ξ*), *V*(*X*, *T*)=*v*_1_(*ξ*), and *c* is the wave speed. Then, the equations (13a) and (13b) are combined as follows:

(14) $$\begin{array}{c} -c\left(u_{1}^{\prime }+v_{1}^{\prime }\right)=v_{1}^{\prime \prime }-k_{1}k_{2}v_{1}-k_{1}v_{1}^{2}+u_{1}-u_{1}^{2}-\left(1-k_{1}\right)u_{1}v_{1}-\left(1-k_{1}\right)u_{1}v_{1}^{2}. \end{array}$$

Following the steps of the algorithm in Section 2, the solutions of *u*_1_ and *v*_1_ can be expressed as follows:

(15) $$\begin{array}{c} u_{1}\left(\xi \right)=\alpha_{1}F\left(\xi \right),\\ v_{1}\left(\xi \right)=\beta_{1}F\left(\xi \right). \end{array}$$

Substituting equation (15) into equation (14) to obtain

(16) $$\begin{array}{c} c\left(\alpha_{1}+\beta_{1}\right)F^{\prime }\left(x\right)+\beta_{1}F^{\prime \prime }\left(x\right)+\left(\alpha_{1}-\beta_{1}k_{1}k_{2}\right)F\left(x\right)+\left(\alpha_{1}\beta_{1}\left(k_{1}-1\right)-\beta_{1}^{2}k_{1}-\alpha_{1}^{2}\right)F^{2}\left(x\right)+\alpha_{1}\beta_{1}^{2}\left(k_{1}-1\right)F^{3}\left(x\right)=0. \end{array}$$

### 3.1. The (*G*′/*G*)-Expansion Method

In order to utilize the (*G*′/*G*)-expansion method, the definition of *F*′ equation (6) is applied to equation (16), and then the following algebraic system is obtained:

(17) $$\begin{array}{c} \alpha_{1}\left(1-c\lambda \right)+\beta_{1}\left(-c\lambda +\lambda^{2}-k_{1}k_{2}+2\mu \right)=0,\\ -\alpha_{1}^{2}-\alpha_{1}\left(c-\beta_{1}\left(k_{1}-1\right)\right)-\beta_{1}\left(c-3\lambda +\beta_{1}k_{1}\right)=0,\\ \beta_{1}\left(\alpha_{1}\beta_{1}\left(k_{1}-1\right)+2\right)=0,\\ -\mu \left(\alpha_{1}c+\beta_{1}\left(c-\lambda \right)\right)=0. \end{array}$$

Hence, two cases of the value of *μ*, *λ*, *c* and *α*_1_ are realized:

- Case I

(18) $$\begin{array}{c} c=\frac{\beta_{1}^{4}{\left(k_{1}-1\right)}^{2}k_{1}+2\beta_{1}^{2}{\left(k_{1}-1\right)}^{2}+4}{2\beta_{1}\left(k_{1}-1\right)\left(\beta_{1}^{2}\left(k_{1}-1\right)-2\right)},\\ \mu =\frac{\beta_{1}^{2}\left(k_{1}-1\right)k_{1}k_{2}+2}{2\beta_{1}^{2}\left(k_{1}-1\right)},\\ \lambda =\frac{\beta_{1}^{4}{\left(k_{1}-1\right)}^{2}k_{1}+2\beta_{1}^{2}{\left(k_{1}-1\right)}^{2}+4}{2\beta_{1}^{3}{\left(\text{k}1-1\right)}^{2}},\\ \alpha_{1}=-\frac{2}{\beta_{1}\left(k_{1}-1\right)}. \end{array}$$

(ii) Case II

(19) $$\begin{array}{c} \mu =0,\\ c=\frac{1}{\beta_{1}-\left(2/\beta_{1}\left(k_{1}-1\right)\right)}\left(-\frac{1}{4}\beta_{1}^{2}k_{1}-\frac{4}{\beta_{1}^{2}{\left(k_{1}-1\right)}^{2}}+\frac{3k_{1}}{\beta_{1}^{2}{\left(k_{1}-1\right)}^{3}}-\frac{3}{\beta_{1}^{2}{\left(k_{1}-1\right)}^{3}}-\frac{3}{4}\beta_{1}\sqrt{\frac{{\left(\beta_{1}^{4}{\left(k_{1}-1\right)}^{2}k_{1}+2\beta_{1}^{2}{\left(k_{1}-1\right)}^{2}+4\right)}^{2}-8\beta_{1}^{4}{\left(k_{1}-1\right)}^{3}\left(\beta_{1}^{2}\left(k_{1}-1\right)k_{1}k_{2}+2\right)}{\beta_{1}^{6}{\left(k_{1}-1\right)}^{4}}}+\frac{3k_{1}}{2\left(k_{1}-1\right)}+\frac{3}{2-2k_{1}}-2\right),\\ \lambda =\frac{1}{8}\left(2\beta_{1}k_{1}+\frac{4k_{1}}{\beta_{1}\left(k_{1}-1\right)}+\frac{8k_{1}}{\beta_{1}^{3}{\left(k_{1}-1\right)}^{3}}-\frac{4}{\beta_{1}\left(k_{1}-1\right)}-\frac{8}{\beta_{1}^{3}{\left(k_{1}-1\right)}^{3}}-2\sqrt{\frac{{\left(\beta_{1}^{4}{\left(k_{1}-1\right)}^{2}k_{1}+2\beta_{1}^{2}{\left(k_{1}-1\right)}^{2}+4\right)}^{2}-8\beta_{1}^{4}{\left(k_{1}-1\right)}^{3}\left(\beta_{1}^{2}\left(k_{1}-1\right)k_{1}k_{2}+2\right)}{\beta_{1}^{6}{\left(k_{1}-1\right)}^{4}}}\right),\\ \alpha_{1}=-\frac{2}{\beta_{1}\left(k_{1}-1\right)}. \end{array}$$

**Figure 1:**
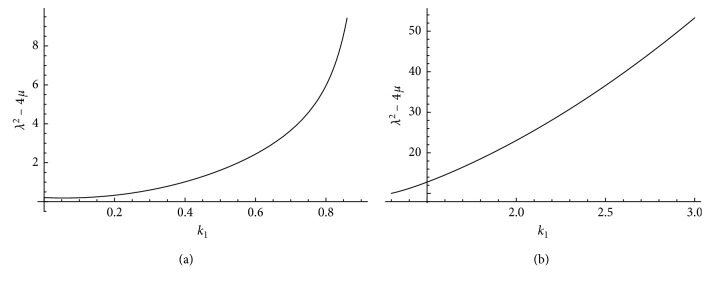
Plot *λ* ^2^ − 4*μ* ^2^ versus *k*_1_ of case (I) where *β*_1_ and *k*_2_ are fixed. (a) $0<k_{1}<,\;\left(0<\sqrt{a_{4}a_{1}}<a_{2}\right)$. (b) $k_{1}>1,\;\left(\sqrt{a_{4}a_{1}}>a_{2}\right)$.

**Figure 2:**
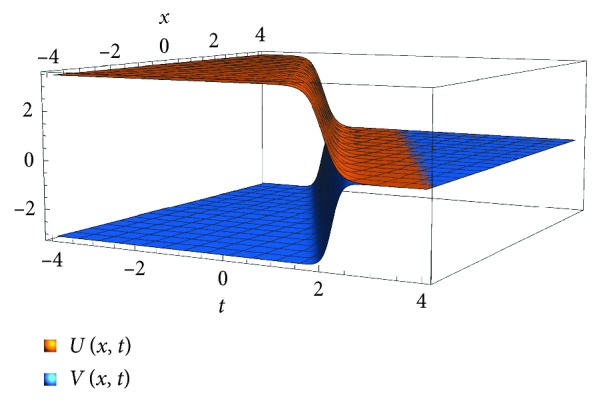
Plot of the scaled density of the prey for *t*=*x*=[−4,4], *μ*=0, *λ*=2(*β*_1_^2^+2)/3*β*_1_, *α*_1_=−*β*_1_, *k*_1_(2/*β*_1_^2^)+1, and *k*_2_=(4*β*_1_^4^+7*β*_1_^2^+16)/(9*β*_1_^2^+18), where *β*_1_=0.5.

The solutions within both cases exist if *k*_1_ ≠ 1. As we see in Figure 1, *λ* ^2^ − 4*μ* > 0 for *k*_1_ ≠ 1. Therefore, the solution of the system is in terms of the hyperbolic function and parameters *k*_1_, *β*_1_ and *k*_2_. Assuming 0 < *k*_1_ < 1 yields $\sqrt{a_{4}a_{1}}<a_{2}$ which is not a reasonable case in actual situations. Therefore, the solution is considered only for *k*_1_ > 1, ($\sqrt{a_{4}a_{1}}>a_{2}$). The following is the solution by the (*G*′/*G*)-expansion method which is a kink soliton solution for *U* and *V* as we see in Figure 2:

(20) $$\begin{array}{c} U\left(\xi \right)=-\frac{2}{\beta_{1}\left(k_{1}-1\right)}\frac{\sqrt{\lambda^{2}-4\mu }}{2}\frac{\left(A_{1}\text{cos }h\left(\left(1/2\right)\sqrt{\lambda^{2}-4\mu }\xi \right)+A_{2}\text{sin }h\left(\left(1/2\right)\sqrt{\lambda^{2}-4\mu }\xi \right)\right)}{\left(A_{1}\text{sin }h\left(\left(1/2\right)\sqrt{\lambda^{2}-4\mu }\xi \right)+A_{2}\text{cos }h\left(\left(1/2\right)\sqrt{\lambda^{2}-4\mu }\xi \right)\right)}-\frac{\lambda }{2},\\ V\left(\xi \right)=\beta_{1}\frac{\sqrt{\lambda^{2}-4\mu }}{2}\frac{\left(A_{1}\text{cos }h\left(\left(1/2\right)\sqrt{\lambda^{2}-4\mu }\xi \right)+A_{2}\text{sin }h\left(\left(1/2\right)\sqrt{\lambda^{2}-4\mu }\xi \right)\right)}{\left(A_{1}\text{sin }h\left(\left(1/2\right)\sqrt{\lambda^{2}-4\mu }\xi \right)+A_{2}\text{cos }h\left(\left(1/2\right)\sqrt{\lambda^{2}-4\mu }\xi \right)\right)}-\frac{\lambda }{2}. \end{array}$$

### 3.2. The Generalized Auxiliary Equation Method

This subsection presents the solution of the considered system by applying the auxiliary equation method. Thus, we use the definition of *F*′ (9) into equation (16) which yields to the following algebraic systems:

(21) $$\begin{array}{c} \alpha_{1}+\beta_{1}=0,\\ \alpha_{1}+\beta_{1}\left(h_{2}-k_{1}k_{2}\right)=0,\\ -\alpha_{1}^{2}+\frac{1}{2}\beta_{1}\left(3h_{3}-2\beta_{1}k_{1}\right)+\alpha_{1}\beta_{1}\left(k_{1}-1\right)=0,\\ \beta_{1}\left(2h_{4}+\alpha_{1}\beta_{1}\left(k_{1}-1\right)\right)=0,\\ \frac{\beta_{1}h_{1}}{2}=0. \end{array}$$

The solutions of the algebraic system aforementioned give *h*_1_=0,  *h*_2_=1+*k*_1_ *k*_2_,  *h*_3_=(4*k*_1_ *β*_1_/3),  *h*_4_=(1/2)(*k*_1_ − 1)*β*_1_^2^, and *h*_0_, *k*_1_, *β*_1_ and *k*_2_ are arbitrary constants. Hence, the appropriate solution of the auxiliary equation is in terms of the hyperbolic function. Assuming *h*_0_=0 yields the solution of the problem in the following expression:

(22) $$\begin{array}{c} U\left(x,t\right)=-\frac{\beta_{1}\left(k_{1}k_{2}+1\right)\sec\;h^{2}\left(\left(1/2\right)\sqrt{k_{1}k_{2}+1}\left(x-ct\right)\right)}{\sqrt{2}\sqrt{\beta_{1}^{2}\left(k_{1}-1\right)\left(k_{1}k_{2}+1\right)}\tan\text{ h}\left(\left(1/2\right)\sqrt{k_{1}k_{2}+1}\left(x-ct\right)\right)-\left(4\beta_{1}k_{1}/3\right)},\\ V\left(x,t\right)=-U\left(x,t\right). \end{array}$$

**Figure 3:**
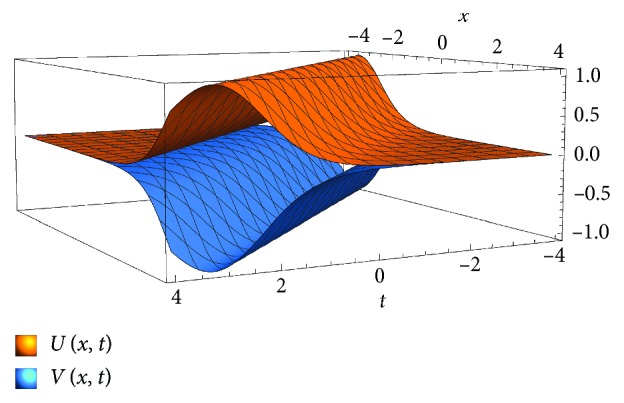
Plot of the scaled density of the prey equations (22) for *t*=*x*=[−4,4], *k*_2_=0.5,  *β*_1_=1, *c*=1, and (*k*_1_=3).

The computed solution by the generalized auxiliary equation method exists if *k*_1_ ≠ 1 and is a soliton solution in terms of *k*_1_, *k*_2_ and *β*_1_ (see Figure 3).

## 4. Discussion

### 4.1. Biological Implication

The solution is obtained by the (*G*′/*G*)-expansion method for $\lambda >2\sqrt{\mu }$ and with the condition *a*_4_ > (*a*_2_^2^/*a*_1_). Thus, this solution is obtained when the decay rate of the prey population is greater due to the interaction between two predators and one prey (*a*_4_) than the decay rate of the prey population due to the competition on the food supply over the growth rate of the prey (*a*_2_^2^/*a*_1_). The solution in Figure 2 shows the predator population increases because of plentiful prey and sexual interaction while the prey population decline because of its high consumption during the mating period of the predator in a close environmental area. Ultimately, the predator population will become dominant in the area. This situation is expected when the predators overgraze in the mating period where the prey is plentiful.

Figure 3 presents the solution by applying the generalized auxiliary equation method for *k*_1_ ≠ 1. This solution depicts that in certain environmental area, the prey population *u* will grow due to the absence of the predator *v*. In the mating period, the predators graze in where the prey density (*u*) is large and as a result *u* will decay. However, the obtained solutions by both considered methods remain constant away from places of grazing predator during the predator-mating period and over time.

### 4.2. Connection with the Previous Studies

The diffusive predator-prey model has been solved numerically and analytically in some papers. The analytical solutions of the model when both species having the same diffusivities and the prey density being of the Allee type are computed by the (*G*′/*G*)-expansion method [16], and the results are either single structure or periodic structure. Also, it was computed by the improved Riccati equation mapping method [17], and the solutions are presented in three structures: single, periodic, and kink. The same model was solved analytically by utilizing the exp-expansion method [18], and the obtained solutions were kink solutions, singular kink solutions, dark soliton solutions, bright soliton solutions, soliton solutions, singular soliton solutions, multiple soliton-like solutions, and triangular periodic solutions, but all these solutions were not discussed in terms of the actual situation.

The numerical solution of a system of nonlinear Volterra differential equations governing on the problem of prey and predator was solved numerically by the Adomian decomposition method [19], Runge–Kutta–Fehlberg method and Laplace Adomian decomposition method [20], and the results state that the number of predator increases, as the number of prey decreases.

The present paper studied the predator-prey model with adding the term *uv* ^2^ and in absence of diffusion among preys *D*_1_=0. We obtained that predator's population increases, as the prey's population decreases in a certain interval, and the solutions are constant out of this interval. Comparing the results obtained in the present study with different modifications of the diffusive predator-prey model, it can be concluded that obtained results are new and different.

## 5. Conclusion

In this paper, both the (*G*′/*G*)-expansion method and the generalized auxiliary equation method have been used to find new stable analytical solutions of the modified nonlinear diffusive predator-prey model by considering the term *uv* ^2^. To the best of the researchers' knowledge, this modified model has not been solved analytically or numerically in previous research. The result showed the effect of the term *uv* ^2^ in the diffusive predator-prey dynamic system which leads to a decline in the prey population and an increase in the predator population in the mating period at predator's density points. However, the idea of this modified system can be used in epidemiological researches to construct several models. For example, if we consider *u* as the infected human body by any disease, in many cases we use two types of medicine which is considered as *v* ^2^. Thus, the interaction term *uv* ^2^ refers to combatting the epidemic or the disease through two types of medicine. In addition, the prey-predator system has been studied widely in the literature regarding the time delay [21–23]. In our future work, we will study time delay for our new modified system with the effect of fluctuation of the biological parameters or fuzzy parameters.

## Figures and Tables

**Table 1:** Description of variables and parameters of model (1).

Variable	Description
*u*=*u*(*x*, *t*)	Density of prey
*v*=*v*(*x*, *t*)	Density of predator
*D*_1_(Δ*u*)	Diffusion term of prey species
*D*_2_(Δ*v*)	Diffusion term of predator species
*D*_1_ and *D*_2_	Diffusion coefficients which are small positive constants
*a*_1_	Growth rate of the prey
*a*_2_	Decay rate of prey due to competition on the food supply between the male and the female
*a*_3_	Decay rate of prey due to the interaction between one predator and one prey
*a*_4_	Decay rate of prey due to the interaction between two predators and one prey
*a*_5_	Mortality rate of predator
*a*_6_	Decay rate of predator due to competition on the food supply between the male and the female

## Data Availability

The data supporting this research are from previously reported studies, which have been cited.
